# Transcatheter Aortic Valve Implantation with ACURATE *neo*: Results from the PROGRESS PVL Registry

**DOI:** 10.1155/2022/9138403

**Published:** 2022-06-25

**Authors:** Won-Keun Kim, Holger Thiele, Axel Linke, Thomas Kuntze, Stephan Fichtlscherer, John Webb, Michael W. A. Chu, Matti Adam, Gerhard Schymik, Tobias Geisler, Rajesh Kharbanda, Thomas Christen, Dominic Allocco

**Affiliations:** ^1^Department of Cardiology and Cardiac Surgery, Kerckhoff Heart Center, Bad Nauheim, Germany; ^2^Department of Cardiology, Heart Center Leipzig at University of Leipzig and Leipzig Heart Institute, Leipzig, Germany; ^3^Department of Internal Medicine and Cardiology, Herzzentrum Dresden at Technische Universität Dresden, Dresden, Germany; ^4^Department of Cardiology, Heart Centre, Central Clinic in Bad Berka, Bad Berka, Germany; ^5^Department of Internal Medicine, Division of Cardiology, Johann W. Goethe University, Frankfurt, Germany; ^6^Centre for Heart Valve Innovation, St. Paul's and Vancouver General Hospitals, University of British Columbia, Vancouver, British Columbia, Canada; ^7^Division of Cardiac Surgery, Department of Surgery, Western University, London Health Sciences Centre, London, Ontario, Canada; ^8^Clinic for Cardiology, University Hospital Cologne, Cologne, Germany; ^9^Medical Clinic IV, Department of Cardiology, Municipal Hospital Karlsruhe, Karlsruhe, Baden-Württemberg, Germany; ^10^Department of Cardiology and Angiology, University Hospital Tübingen, Tübingen, Germany; ^11^Oxford Heart Centre, John Radcliffe Hospital, Oxford, UK; ^12^Boston Scientific, Marlborough, MA, USA

## Abstract

**Objectives:**

The PROGRESS PVL registry evaluated transcatheter aortic valve implantation (TAVI) in patients treated with ACURATE *neo*, a supra-annular self-expanding bioprosthetic aortic valve.

**Background:**

While clinical outcomes with TAVI are comparable with those achieved with surgery, residual aortic regurgitation (AR) and paravalvular leak (PVL) are common complications. The ACURATE *neo* valve has a pericardial sealing skirt designed to minimize PVL.

**Methods:**

The primary endpoint was the rate of total AR over time, as assessed by a core echocardiographic laboratory. The study enrolled 500 patients (mean age: 81.8 ± 5.1 years; 61% female; mean baseline STS score: 6.0 ± 4.5%) from 22 centers in Europe and Canada; 498 patients were treated with ACURATE *neo*.

**Results:**

The rate of ≥ moderate AR was 4.6% at discharge and 3.1% at 12 months; the rate of ≥ moderate PVL was 4.6% at discharge and 2.6% at 12 months. Paired analyses showed significant improvement in overall PVL between discharge and 12 months (*P* < 0.001); 64.6% of patients had no change in PVL grade, 24.9% improved, and 10.5% worsened. Patients also exhibited significant improvement in transvalvular gradient (*P* < 0.001) and effective orifice area (*P*=0.01). The mortality rate was 2.2% at 30 days and 11.3% at 12 months. The permanent pacemaker implantation (PPI) rate was 10.2% at 30 days and 12.2% at 12 months.

**Conclusions:**

Results from PROGRESS PVL support the sustained safety and performance of TAVI with the ACURATE *neo* valve, showing excellent valve hemodynamics, good clinical outcomes, and significant interindividual improvement in PVL from discharge to 12-month follow-up.

## 1. Introduction

Transcatheter aortic valve implantation (TAVI) is an established, effective alternative treatment for patients with symptomatic aortic stenosis who are considered high-risk for surgical valve replacement. While clinical outcomes with TAVI are comparable to those achieved with surgery, concerns over complications such as residual aortic regurgitation (AR) and paravalvular leak (PVL) persist. Moderate or greater PVL has been linked to less robust functional improvement, increased rates of heart failure and hospitalization, and increased long-term mortality [1–3].

The ACURATE *neo* valve, a self-expanding, supra-annular bioprosthetic aortic valve, has been commercially available for transfemoral TAVI in Europe since 2014. The largest study of ACURATE *neo* to date, the SAVI-TF study, investigated clinical and echocardiographic outcomes in a large high-risk patient population treated under real-world conditions [4, 5]. The study had a 98.7% procedural success rate, a low rate of all-cause mortality (30 days: 1.4%; 1 year: 8.0%), and the pacemaker rate was <10% through 1 year. Patients exhibited a low rate of ≥ moderate PVL at 30 days (4.1%) and 1 year (3.8%). However, other studies of ACURATE *neo* have produced conflicting data regarding PVL. Patients randomized to treatment with ACURATE *neo* in the SCOPE I and SCOPE II studies had a higher incidence of ≥ moderate PVL at 30 days compared with Sapien 3 and Evolut R/PRO, respectively, which contributed to ACURATE *neo* missing the noninferiority primary endpoints in both studies [6, 7].

Here, we report results from the PROGRESS PVL registry, which evaluated the safety and performance of TAVI with ACURATE *neo* in routine clinical practice. The study also included longitudinal assessment of echocardiographic data over time by an independent core laboratory.

## 2. Methods

### 2.1. Study Design

PROGRESS PVL is a multicenter open-label single-arm study. Patients were considered eligible if they presented with severe aortic stenosis and were determined by a heart team to be at high risk for surgical valve replacement based on the patient's Society of Thoracic Surgeons (STS) score as well as the presence of other comorbidities. The protocol was approved by locally appointed institutional review boards/ethics committees. The study was conducted in accordance with the International Conference for Harmonization Good Clinical Practice (ICH-GCP) regulations and guidelines and the ethical principles outlined in the Declaration of Helsinki and registered with ClinicalTrials.gov (NCT02987894). All patients gave written informed consent.

### 2.2. Device Details

**Figure 1:**
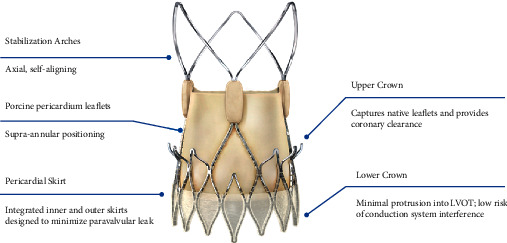
The ACURATE *neo* valve. The ACURATE *neo* valve is comprised of three porcine pericardial leaflets sewn into a self-expanding nitinol frame with three axial stabilization arches. The supra-annular positioning contributes to low gradients. The pericardial skirt is designed to minimize paravalvular leak.

Figure 1 presents key elements of the ACURATE *neo* valve (Boston Scientific, Marlborough, MA, USA), which have been described in detail elsewhere [8]. The valve features controlled and predictable top-down deployment, with sequential release of the stabilization arches followed by the upper and lower crown, allowing for hemodynamic stability and uninterrupted aortic outflow. ACURATE *neo* is available in three sizes (S [small], M [medium], and L [large]) to treat native annulus diameters of 21 mm to 27 mm. Valve sizing was assessed by computerized tomography (CT) and based on perimeter-derived annulus diameter; final size selection was at the operators' discretion.

### 2.3. Outcomes Measures and Statistical Methods

The primary endpoint was the rate of total aortic regurgitation (AR) at discharge/7 days, 30 days, and 12 months after TAVI. The degree of paravalvular leak (PVL) was also examined, as this is the main driver of AR after TAVI and is typically of greater clinical interest. Per protocol, echocardiographic assessments were carried out according to local standard of care for TAVI (if frequency or requirements were different from the study schedule) with all available data assessed by an independent core laboratory (Medical Research Development, Madrid, Spain). The primary endpoint and all echocardiographic outcomes, including improvements in mean transvalvular gradient and effective orifice area (EOA), were measured in the per-protocol population (i.e., patients treated with the ACURATE *neo* valve). Clinical event rates were analyzed in the intent-to-treat (ITT) population, which includes all enrolled patients in whom valve implantation was attempted. All VARC-2 safety events were evaluated by an independent medical reviewer. A comprehensive list of secondary endpoints is presented in Supplementary Table 1. A post-hoc analysis was performed to compare the size of the implanted prosthetic valve with the native annulus dimensions (based on site-reported CT), expressed as cover index [CI = 100 x (nominal prosthesis diameter—annulus diameter)/nominal prosthesis diameter].

Baseline and outcome variables were summarized using descriptive statistics. For comparison of categorical variables, statistical differences were assessed using a chi-square test or a Fisher's exact test, as appropriate. For comparison of continuous variables, the Student's *t*-test or analysis of variance was used. Paired analysis of change in PVL over time was performed according to Bhapkar's test for marginal homogeneity. All statistical analyses were two-sided with an alpha level of 5%. Statistical analyses were performed with SAS software (SAS Institute Inc., Cary, NC), version 9.3 or later.

## 3. Results

### 3.1. Study Cohort

**Figure 2:**
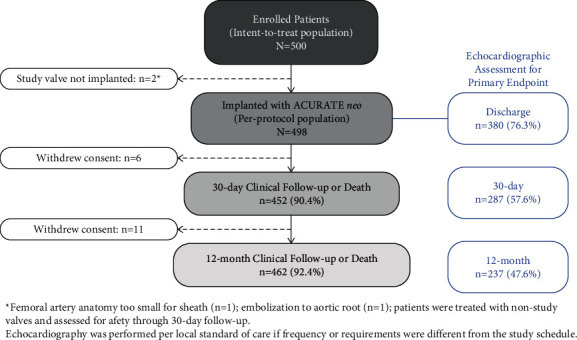
PROGRESS PVL study flow.

PROGRESS PVL enrolled 500 patients at 22 centers in Europe and Canada between January 2017 and July 2018. A listing of investigators and sites can be found in Supplementary Table 2.  Figure 2 depicts the disposition of enrolled patients. Two patients were not implanted with ACURATE *neo* and thus were not included in the per-protocol analysis set. In one patient, the femoral artery anatomy was too small for the delivery system sheath, and in another patient, the valve lost contact with the annulus and embolized to the aortic root (valve was snared in the ascending aorta); these patients were treated with nonstudy valves and assessed for safety through 30-day follow-up.

The mean age of enrolled patients was 81.8 ± 5.1 years, and 61.2% were female. The mean STS score in the study population was 6.0 ± 4.5%, and 24.4% of patients had an STS score ≥8%. At baseline, New York Heart Association (NYHA) functional status was class III or IV in 75.2% of patients. Based on site-reported assessment, calcification of the aortic leaflets was severe or extreme in approximately one-third of patients. Additional baseline demographics, risk factors, and preexisting clinical conditions are detailed in Supplementary Table 3.

### 3.2. Procedural Details

Procedural characteristics are summarized in Table 1. Preimplant balloon aortic valvuloplasty was performed in 91.4% of patients; postdilatation was performed in 45.1% of patients. Most patients were implanted with either a M (38.8%) or L (41.6%) valve. The median cover index in the as-treated population was 5.6%. Correct positioning of a single valve in the proper location occurred in 493/500 patients (98.6%). In addition to the two cases previously described, who were not treated with an ACURATE *neo* valve, there were four valve-in-valve procedures treated with a balloon-expandable nonstudy valve (two cases where the initial ACURATE bioprosthesis was placed in the aortic root but then lost contact with the annulus, one case of dislocation to aortic root with incomplete valve expansion, and one case of dislocation into the left ventricular outflow tract) and one case where the initial valve was malpositioned with no further action noted. Patients treated with nonstudy valves in a valve-in-valve procedure were assessed for clinical safety events but were not included in the per-protocol analysis set for echocardiographic outcomes. In the case wherein the initially implanted ACURATE *neo* valve lost contact with the annulus, implantation with a nonstudy valve was attempted, but the patient subsequently experienced cardiogenic shock, resulting in death the same day as the index procedure, for a procedural mortality rate of 0.2%. There were no instances of conversion to surgery, annular rupture, or ventricular septal perforation. One patient experienced dissection of the ascending aorta, with subsequent endocarditis reported at three months after index procedure.

### 3.3. Echocardiographic Outcomes

**Figure 3:**
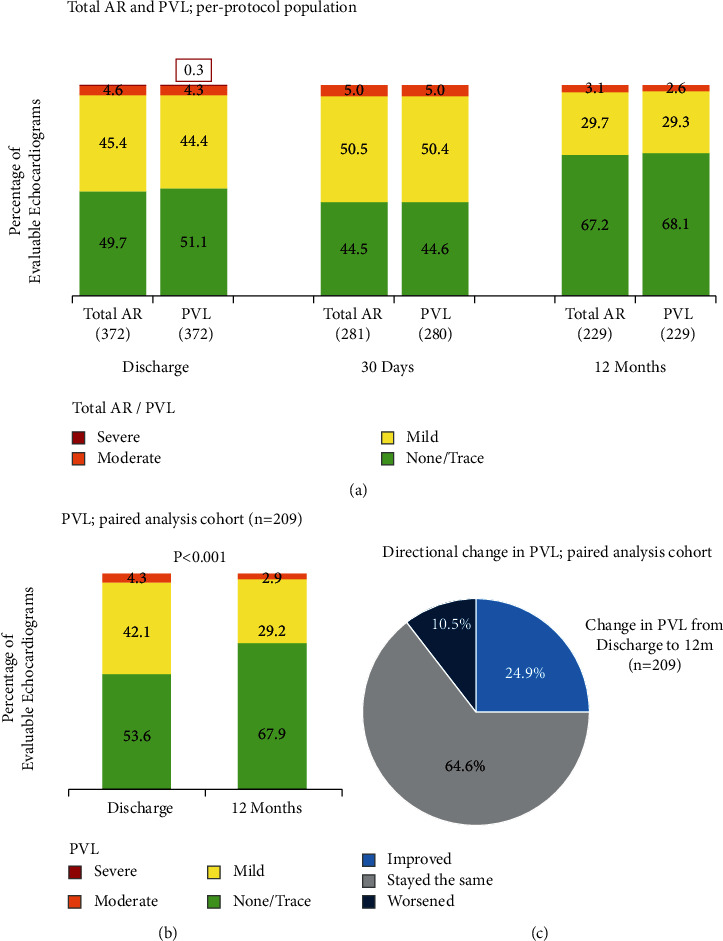
Improvement in aortic regurgitation. (a) The primary endpoint of PROGRESS PVL was the rate of total aortic regurgitation (AR), assessed by an independent echocardiography laboratory at discharge/7 days, 30 days, and 12 months in patients treated with ACURATE *neo*. Paravalvular leak (PVL) was very similar to total AR at all time points. (b, c) Paired analyses performed in patients with data available at both discharge and 12 months (*N* = 209) demonstrated significant overall improvement in PVL (*P* < 0.001; Bhapkar's test for marginal homogeneity), with a greater proportion of patients showing interindividual improvement in PVL compared with worsening PVL. All echocardiographic data were assessed by a core laboratory.

In the overall population, ≥ moderate AR was observed in 4.6% of patients at discharge and 3.1% at 12 months (Figure 3(a)). Observed PVL was very similar to total AR at all time points, with ≥ moderate PVL in 4.6% of patients at discharge and 2.6% at 12 months (Figure 3(a)). The median cover index was significantly higher in patients with no/trace PVL at discharge compared with patients with mild or greater PVL (6.7% vs 4.8%; *P* < 0.001) (Supplementary Figure 1). A paired analysis performed among patients with core laboratory-adjudicated echo data available at discharge and 12 months (*n* = 209) demonstrated significant overall improvement in PVL (*P* < 0.001; Figure 3(b)). The proportion of patients with improved PVL between discharge and 12 months was greater than that with worsening PVL (24.9% vs. 10.5%) (Figure 3(c)).

**Figure 4:**
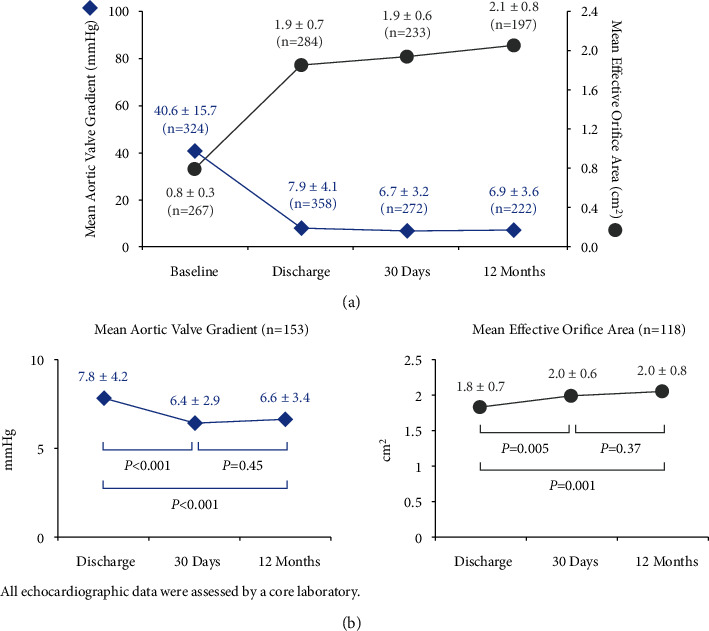
Change in valve hemodynamics. (a) Patients treated with ACURATE *neo* demonstrated improvements in mean aortic valve gradient and mean effective orifice area through 12-month follow-up, as assessed by an independent echocardiography laboratory. (b) Paired analyses of core laboratory data at discharge, 30 days, and 12 months demonstrate early hemodynamic improvement on a per-subject basis, which was maintained through 12 months.

Patients treated with ACURATE *neo* demonstrated improved valve hemodynamics through 12 months of follow-up. In the per-protocol population, the mean aortic valve gradient declined and mean EOA increased substantially from baseline to discharge (Figure 4(a)). Improvements in gradient and EOA were observed across valve sizes (Supplementary Figure 2). Paired analyses performed in patients with hemodynamic data available at discharge, 30 days, and 12 months demonstrate maintenance of significant improvement between discharge and 12 months in transvalvular gradient (*P* < 0.001) and EOA (*P*=0.01) (Figure 4(b)).

### 3.4. Clinical Safety and Functional Improvement

Clinical safety outcomes were analyzed for all enrolled patients in whom valve implantation was attempted (i.e., the ITT population) and are presented in Table 2. The VARC-2 composite endpoint for early safety at 30 days was met by 9.2% of patients, with low rates of all-cause mortality and disabling stroke at 30 days. The overall rate of permanent pacemaker implantation (PPI) was 10.2% at 30 days and 12.2% at 12 months (11.6% and 13.4%, respectively, in patients who did not have a pacemaker at baseline). There were no instances of coronary obstruction during the study. The rate of prosthetic valve thrombosis was very low, with only one case occurring within 12 months.

**Figure 5:**
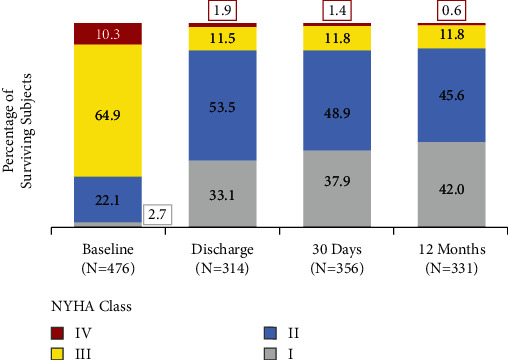
Improvement in functional status. Improvement in functional status, based on New York heart association (NYHA) functional class, was evident at discharge and sustained through 12 months of follow-up. Analysis includes surviving subjects treated with ACURATE neo who had functional status recorded at a given time point.

Figure 5 illustrates the functional status of patients treated with ACURATE *neo* (per-protocol population) based on NYHA functional class at baseline, discharge, 30 days, and 12 months after the procedure. At discharge, 86.6% of patients evaluated were class I or II, with 71.7% of patients showing improvement from baseline of at least one class, and 25.2% showing improvement of at least two classes. This trend continued, with 87.6% of surviving patients classified as class I or II at 12 months. One year after TAVI, 76.4% and 33.8% of patients had improved at least one or two classes from baseline, respectively.

## 4. Discussion

PROGRESS PVL represents an extension of the body of evidence supporting TAVI with ACURATE *neo,* providing real-world data in an elderly high-risk patient population. Importantly, the study includes longitudinal echocardiographic data adjudicated by an independent core laboratory.

Patients maintained excellent valve hemodynamics, with large EOAs and low gradients, as expected for a supra-annular valve. Moderate or greater PVL was 2.6% at 1 year, which is lower than observed in earlier ACURATE *neo* studies (SAVI-TF: 3.6%, CE-mark cohort: 4.5%) [5, 9]. In the paired analysis, patients showed significant overall improvement in PVL from discharge to 12-month follow-up, with 64.6% of patients showing no change and 24.9% showing improvement in PVL grade over time. There is some evidence that self-expanding prostheses have the potential for continued frame expansion and adaptation to the annulus, thus contributing to a reduction in PVL over time [10]. The results with ACURATE *neo* are consistent with data from the Italian CoreValve registry, in which all patients with mild leak after procedure were either unchanged or improved through three years of follow-up [11], and the CoreValve U.S. Pivotal Trial, which noted improvement in the severity of PVL grade in patients with paired discharge and one-year echocardiograms [12]. It is less clear whether PVL also continues to improve following TAVI with a balloon-expandable valve. In the PARTNER study, PVL was unchanged through two-year follow-up in 46.2% of patients treated with a Sapien valve, improved in 31.5%, and worsened in 22.4% [13], while a paired analysis of patients treated with Sapien 3 in the PARTNER 2A study did not show any difference in PVL between 30-day and one-year follow-up [14].

The rate of procedural complications was low in PROGRESS PVL, with correct positioning of the valve in 98.6% of patients, and there were no cases of coronary obstruction. Many of the participating centres and investigators in the current study had prior experience with ACURATE *neo*, which likely increased their comfort with using the valve, leading to fewer procedural complications. The 30-day PPI rate in the present study (10.2%) was in the range of that observed in other studies of ACURATE *neo* (SAVI-TF: 8.3%, SCOPE I: 10.0%, SCOPE II: 11.0%) [5–7]. It is possible that variations in PPI may be attributed to differences in positioning strategies. A recent publication comparing PPI rate in patients categorized by their consecutive enrolment in a large European registry found that as positioning strategy evolved from a low implantation depth to a deliberately higher position, PPI rate was reduced (quartile 1–3: 10.9% vs. quartile 4 : 7.4%) [15]. Prior experience with implanting ACURATE *neo*, including consideration of positioning, may have had an impact on the PPI rate in PROGRESS PVL; however, collection of detailed imaging data to allow for an in-depth analysis of positioning was outside the scope of the current study.

Early clinical outcomes with ACURATE *neo* were similar to or better than those observed with other first-generation valves. The 30-day rates for all-cause mortality (2.2%) and stroke (2.6%) are comparable to the ranges observed in studies of Sapien/Sapien XT (3.5%–6.3% and 3.6%–5.8% , resp.) [16–19] or CoreValve (2.1%–5.1% and 1.4%–4.9%, resp.) [16, 20–22]. However, contextualizing the data from PROGRESS PVL is difficult, due in part to differences in patient risk assessments across studies. Also, as the practice of TAVI has evolved and operators have become more experienced, implant technique and patient selection have become more refined, leading to an overall decline in procedural complications and improved early outcomes [23, 24].

The investigator-initiated SCOPE I and II studies have presented head-to-head comparisons of ACURATE *neo* with later generation competitor devices. In SCOPE I, ACURATE *neo* did not meet the noninferiority criteria vs. Sapien 3 for the 30-day composite endpoint, partially driven by a higher rate of PVL (9.4% vs. 2.8%; *P* < 0.001) [6]. However, at one-year follow-up, clinical and functional outcomes did not differ significantly between the devices, and the mean AV gradient was significantly lower and median EOA significantly larger in patients treated with ACURATE *neo* compared with those treated with Sapien 3 [25]. ACURATE *neo* also missed the noninferiority composite endpoint of all-cause death or any stroke at 30 days vs. Evolut R/PRO in SCOPE II [7], which was attributed to a higher rate of cardiac mortality (3% vs 1%, resp.; *P*=0.03). The rate of cardiovascular death among PROGRESS PVL patients was 2.0% at 30 days. More extensive operator experience with ACURATE *neo* in the current study compared with SCOPE II may have played a factor in the differences in cardiac mortality between these studies.

In the PROGRESS PVL study, the 30-day rate of ≥ moderate PVL (5.0%) was nearly half that observed with ACURATE *neo* in SCOPE I (9.4%) and SCOPE II (9.6%) [6, 7]. The lower PVL rates observed in PROGRESS PVL may be partially explained by operators' prior experience implanting ACURATE *neo* and more careful patient selection. A similar “learning curve effect” has been observed in retrospective analyses of ACURATE *neo*, with substantial improvement in PVL in later cases compared with earlier cases [26, 27]. Through a combination of better patient selection, careful sizing, consideration of aortic valve calcification, and a modified implant technique, it was demonstrated that the rate of ≥ moderate PVL could be reduced to <1% [27]. Additionally, a greater percentage of patients in the current study had size L valves implanted compared with the SCOPE I and II studies (41.6% vs. 34.1% and 33.9%, resp.), indicating a tendency towards oversizing. The median cover index in the PROGRESS PVL as-treated population was 5.6%, and patients with no/trace PVL were found to have a higher median cover index compared with patients with ≥ mild PVL, suggesting oversizing may minimize leak risk. Previous studies have likewise noted an inverse correlation between cover index and annulus diameter and an association between low cover index and mild or greater PVL [28, 29]. Finally, as ACURATE *neo* has a relatively low radial strength, postdilatation may have been used to achieve optimal expansion of the valve and reduce leaks, particularly in annuli that were highly calcified or irregular in shape.

The next iteration of the ACURATE valve, *neo*2, was designed to address concerns related to PVL while maintaining the desirable features of the ACURATE platform. Design enhancements include radiopaque markers that help to facilitate precise valve positioning, and an augmented pericardial sealing skirt, which extends to the waist of the valve and is approximately 60% larger than the prior skirt, to further minimize PVL. In the ACURATE neo AS study, 97% of patients treated with ACURATE *neo*2 exhibited ≤ mild PVL [30]. No patient had severe PVL, and the 30-day rate of moderate PVL (3.0%) was comparable to that observed with Sapien 3 by Mauri et al. (3.6%) and in SCOPE I (2.8%), and with Evolut in SCOPE II (2.9%) [6, 7, 31]. These results are corroborated by preliminary unpublished data from the Early Neo2 Registry, an investigator-initiated registry of >500 European patients treated with *neo2*. The incidence of > mild PVL in the Early Neo2 Registry was 1.3% [32], and a separate core laboratory analysis found that mean aortic regurgitation fraction was significantly lower in patients treated with ACURATE *neo2* versus ACURATE *neo* (4.4 ± 4.8% vs. 9.9 ± 8.2%; *P* < 0.001), as was the rate of moderate/severe aortic regurgitation (1.7% vs. 13.9%; *P* < 0.001) [33].

The impact of PVL on long-term prognosis is an important factor that must be considered, particularly as TAVI is extended to younger and lower-risk patients, for whom long-term valve performance is critical. While moderate-to-severe PVL has consistently been associated with increased mortality, data on the impact of mild PVL on survival is less clear [3, 34, 35]. The current study, which used the standard 3-class grading scheme for PVL, may have overlooked discrete differences between mild and mild-to-moderate PVL, and although these subtle differences could potentially be associated with an adverse effect, the effect size may have been small or masked by several covariates that affect survival. It should also be considered that there are factors that may affect patients' susceptibility to residual PVL (e.g., reduced LV-function; mixed aortic valve disease). Thus, it will be important to minimize even mild PVL, as there could be an impact in some patient subgroups. While our analyses did not identify any factors strongly related to improvement or worsening of PVL, there could be a signal that the presence of eccentric calcification plays a role regarding the potential for improvement of PVL over time. This is likely to be a topic of interest in future studies.

The ACURATE *neo2* design incorporates improved sealing to further reduce PVL, while preserving many desirable features of the ACURATE *neo* platform, including supra-annular valve positioning to allow for low gradients and a simplified implant technique. The stent configuration of the valve remains the same, with a relatively low radial force, which can be expected to contribute to a low pacemaker rate. This expectation is supported by data from the Early Neo2 Registry, in which the in-hospital rate of new PPI was 6.0% [33].

### 4.1. Study Limitations

Interpretation of data from the PROGRESS PVL study is limited by the lack of direct comparison with other devices. As an observational registry, patient selection was not restricted; however, as this was a postmarket study, it is likely that operators had some prior experience with the valve and may have paid more careful attention to patient selection in terms of optimized sizing and avoiding unfavorable calcification patterns (e.g., heavy calcification in the device landing zone), which may have contributed to the low PVL rates observed. There was no core laboratory for CT during initial patient assessment and valve sizing and no formal assessment of cerebrovascular events by a neurological professional, which may have resulted in an underestimation of the stroke rate. As is often the case with patient registries, monitoring was limited, and the rates of clinical and echocardiographic follow-up were relatively low. Geographic variations in standards and frequency of data collection were likely compounded by difficulties in compelling patients to return to sites for scheduled follow-up assessments.

## 5. Conclusions

Patients in the PROGRESS PVL registry demonstrated a lower rate of moderate or greater PVL than has been observed in earlier studies of the ACURATE *neo* valve, with significant interindividual improvement in PVL from discharge to 12-month follow-up. Patients also maintained excellent valve hemodynamics and demonstrated good clinical outcomes overall. The one-year results from this study support the safety and performance of TAVI with ACURATE *neo* in patients with severe aortic valve stenosis and suggest that ACURATE *neo* can be used in any patient that meets the appropriate sizing range, absent in the presence of extreme, or very eccentric aortic valve calcification. The design of the next-generation ACURATE *neo2* valve, which retains many of the desirable features of ACURATE *neo*, allows for improved sealing and thus reduced rates of PVL, conferring potential use in a wider range of patients, including those with irregular or more calcified anatomies.

## Figures and Tables

**Table 1:** Procedural outcomes.

Variable	ITT population (*N* = 500)
Total time from first puncture to time of transfemoral access site closure (min)	56.7 ± 26.8 (499)
Total time from insertion of delivery system to removal of delivery system (min)	10.2 ± 11.6 (491)
Valve size implanted^*∗*^	
S	19.6% (98)
M	38.8% (194)
L	41.6% (208)
Balloon predilatation	91.4% (457)
Postdilatation	45.2% (226)
Correct positioning of a single valve in the proper location	98.6% (493)
Procedural mortality^†^	0.2% (1)
Periprocedural myocardial infarction (≤72 h)^‡^	0.8% (4)
Major vascular complications	2.8% (14)
Life-threatening/disabling bleeding	0.8% (4)
Valve-in-valve implant^^^	0.8% (4/500)
Surgical aortic valve replacement	0.0% (0/500)
Unplanned use of cardiopulmonary bypass	0.0% (0/500)
Coronary obstruction requiring intervention	0.0% (0/500)
Ventricular septal perforation	0.0% (0/500)
Cardiac tamponade	0.0% (0/500)
Endocarditis	0.0% (0/500)
Valve embolization	0.2% (1/500)
Valve thrombosis	0.0% (0/500)

Data are % (*n*) or mean ± standard deviation (*n*). ^*∗*^ Two patients from the ITT population were not implanted with ACURATE *neo*. ^†^ ACURATE *neo* valve lost contact with the annulus; patient was treated valve-in-valve with a nonstudy valve, experienced cardiogenic shock, and died the same day as the index procedure. ^‡^Intra-procedural myocardial infarction, *n* = 2 (STEMI, n = 1; NSTEMI, *n* = 1).

**Table 2:** Clinical safety outcomes.

Variable	30 days	12 months
VARC-2 composite early safety	9.2% (46)	—
^*∗*^All-cause mortality	2.2% (11)	11.3% (54)
Cardiovascular death	2.0% (10)	7.1% (34)
Noncardiovascular death	0.2% (1)	4.2% (20)
^*∗*^Stroke	2.6% (13)	3.6% (17)
Disabling Stroke	2.4% (12)	3.1% (15)
Nondisabling Stroke	0.2% (1)	0.4% (2)
^*∗*^Major vascular complications	3.6% (18)	4.0% (19)
^*∗*^Bleeding, life-threatening or disabling	1.4% (7)	3.4% (16)
Myocardial infarction (>72 h postprocedure)	0.0% (0)	1.0% (5)
^*∗*^Acute kidney injury (AKI stage 2/3)	0.8% (4)	1.0% (5)
New permanent pacemaker implantation		
All patients	10.2% (51)	12.2% (58)
Pacemaker-naive patients (*n* = 443)	11.6% (51)	13.4% (57)
New onset of atrial fibrillation/flutter	5.2% (26)	7.5% (36)
Valve malpositioning^†^	1.4% (7)	1.5% (7)
^*∗*^Coronary obstruction requiring intervention	0.0% (0)	0.0% (0)
Ventricular septal perforation	0.0% (0)	0.0% (0)
Cardiac tamponade	0.0% (0)	0.0% (0)
^*∗*^Repeat procedure for valve-related dysfunction	1.2% (6)	1.7% (8)^‡^
Prosthetic valve endocarditis	0.0% (0)	0.8% (4)
Prosthetic valve thrombosis	0.0% (0)	0.2% (1)

Data are % (*n*), reported for the ITT population (*N* = 500). ^*∗*^ Component of VARC-2 composite endpoint for early safety at 30 days. ^†^ Includes valve migration, valve embolization, ectopic valve deployment; ^‡^Two patients were treated with a repeat procedure after 30 days. In one patient, the 30-day follow-up TEE revealed reduced LVEF with persistent moderate PVL; balloon valvuloplasty was performed but did not improve aortic valve insufficiency, and patient underwent SAVR. One patient experienced endocarditis and associated dissection of the ascending aorta on day 89 post-TAVI; SAVR was performed to replace the ACURATE *neo* valve.

## Data Availability

The data and study protocol for this clinical trial may be made available to other researchers in accordance with Boston Scientific's Data Sharing Policy (https://www.bostonscientific.com/en-US/data-sharing-requests.html).

## Supplementary Materials

Supplementary Materials Supplementary Table 1: primary and secondary endpoints. Supplementary Table 2: investigators and sites by enrolment. Supplementary Table 3: baseline demographics and clinical characteristics. Supplementary Figure 1: prosthetic valve cover index. Supplementary Figure 2: hemodynamic results by valve size.
