# Supplementary material for: Transcatheter Aortic Valve Implantation with ACURATE neo: Results from the PROGRESS PVL Registry
Source: J Interv Cardiol. 2022 Jun 25;2022:9138403. doi: 10.1155/2022/9138403 (PMC9252754; doi:10.1155/2022/9138403)

Supplementary Materials for

“Transcatheter Aortic Valve Implantation with ACURATE *neo*:
Results from the PROGRESS PVL registry”

Won-Keun Kim, MD; Holger Thiele, MD; Axel Linke, MD; Thomas Kuntze, MD; Stephan Fichtlschere^5^, MD; John G Webb, MD; Michael W A Chu, MD; Matti Adam, MD; Gerhard Schymik, MD; Tobias Geisler, MD; Rajesh Kharbanda, MD, PhD; Thomas Christen, MD; Dominic J Allocco, MD

Contents

[Supplementary Table 1. Primary and Secondary Endpoints 2](#_Toc96524017)

[Supplementary Table 2. Investigators and Sites by Enrolment 4](#_Toc96524018)

[Supplementary Table 3. Baseline Demographics and Clinical Characteristics 6](#_Toc96524019)

[Supplementary Figure 1. Prosthetic Valve Cover Index 8](#_Toc96524020)

[Supplementary Figure 2. Hemodynamic Results by Valve Size 9](#_Toc96524021)

## Supplementary Table 1. Primary and Secondary Endpoints

| **Primary Endpoint** | The primary endpoint is total aortic regurgitation post-procedure, at 7 days or discharge (whichever occurs first), 30 days and 12 months follow-up. |
| --- | --- |
| **Secondary Endpoints** | 1. Incidence of all-cause mortality at 30 days post-index procedure. 2. Clinical events as defined per VARC-2 consensus document^1^ (VARC-2) at procedure, 7 days or discharge (whichever occurs first), 30 days and at 12 months:    - All-cause mortality    - All stroke    - Myocardial infarction    - Bleeding complication    - Acute kidney injury    - Vascular complication    - Conduction disturbances and arrhythmias    - Other TAVI-related complications 3. Procedural success defined as absence of intra-procedure mortality and complications arising during implantation of the prosthetic valve such as: inability to properly seat the valve in the annulus; need for more than one implanted aortic bioprosthesis (valve-in-valve or ectopic deployment) or if a surgical aortic valve replacement is required to correct a severe aortic regurgitation or procedure complication. The procedure can be considered as success despite the presence of residual aortic regurgitation which may be due to the anatomic configuration of the annulus or a calcific valvular annulus. 4. Device success at 7-days or discharge (whichever occurs first) defined as:    - Absence of intra-procedure mortality (procedure to 24H) AND,    - Correct positioning (placement in the annulus with no impairment of aortic bioprosthesis function) of a single prosthetic heart valve into the proper anatomical location AND,    - Intended performance of the prosthetic heart valve:      - No prosthesis-patient mismatch (EAOi >0.85 cm^2^/m^2^) AND,      - Mean aortic valve gradient <20mmHg or peak velocity < 3 m/s AND,      - No moderate or severe prosthetic valve regurgitation.   In evaluating echo parameters, values at 7D/Discharge (whichever occurs first) will be used for each of the echo parameters above. If any of echo parameters is missing at 7-days or discharge, post-procedure data may be used for the missing values. If device success or failure cannot be determined due to missing of parameters listed above or un-evaluable echocardiography assessment, device success will be considered not obtainable.   1. VARC-2 Composite Safety at 30-days. 2. Functional improvement from baseline as per NYHA Functional Classification at 7 days or discharge (whichever occurs first), 30 days and 12 months follow-up. 3. Improvement from baseline of the haemodynamic function: effective orifice area and mean transprosthetic gradient at 7 days or discharge (whichever occurs first), 30 days and 12 months follow-up; 4. Valve related dysfunction (7-days/discharge (whichever occurs first), 30-days and12 months) defined as: mean aortic valve gradient ≥ 20mmHg, EOA ≤0.9-1.1 cm2, and/or DVI< 0.35, and/or moderate or severe prosthetic valve regurgitation (See VARC-2 and Figure 4 from VARC manuscript). |
| Kappetein, et al. *European Heart Journal* (2012) 33, 2403–2418. doi:10.1093/eurheartj/ehs255 | |

## Supplementary Table 2. Investigators and Sites by Enrolment

| **Site Principal Investigator** | **Institute** | **City, Country** | **Patients Implanted** |
| --- | --- | --- | --- |
| Holger Thiel | Leipzig Heart Center | Leipzig, DE | 82 |
| Thomas Kuntze | Zentralklinik Bad Berka GmbH | Bad Berka, DE | 66 |
| Stephan Fichtlscherer | Universitätsklinikum Frankfurt | Frankfurt Am Main, DE | 62 |
| Won-Keun Kim | Kerckhoff-Klinik GmbH | Bad Nauheim, DE | 47 |
| John Webb | Providence Health Care Heart Center - St. Paul Hospital | Vancouver, British Columbia, CA | 32 |
| Michael Chu | London Health Science Center | London, Ontario, CA | 31 |
| Matti Adam (Tanja Rudolph at study initiation) | Kardiologie Uniklinik Köln | Köln, DE | 29 |
| Gerhard Schymik | Städisches Klinikum Karlsruhe GmbH | Karlsruhe, DE | 28 |
| Tobias Geisler | Medizinische Universitätsklinik | Tübingen, DE | 24 |
| Rajesh Kharbanda | John Radcliffe Hospital – Oxford University Hospitals | Oxford, UK | 23 |
| Holger Nef | Universitätsklinikum Gießen -Medizinische Klinik und Poliklinik | Giessen, DE | 17 |
| Michael Haude | Städtische Kliniken Neuss - Lukaskrankenhaus GmbH | Neuss, DE | 15 |
| Lars Conzelmann | Helios Klinik fur Herzzchirurgie | Karlsruhe, DE | 10 |
| Sergio Berti | Fondazione Toscana G. Monasterio, Ospedale del Cuore G. Pasquinucci | Massa, IT | 9 |
| Helge Möllmann | St. Johannes Hospital Dortmund | Dortmund, DE | 7 |
| Arnaldo Poli | Ospedale Civile Di Legnano | Legnano, IT | 5 |
| Axel Harnath | Sana-Herzzentrum Cottbus GmbH | Cottbus, DE | 4 |
| Christian Thilo | Klinikum Augsburg | Ausgburg, DE | 2 |
| Jan Kovac | University Hospital of Leicester NHS Trust | Leicester, UK | 2 |
| Florian Hopfner (Hendrick Treede at study initiation) | Universitätsklinikum Halle Universitätsklinik und Poliklinik für Herzchirurgie | Halle, DE | 2 |
| Helmut Baumgartner | Universitätsklinikum Münster, Department für Kardiologie und Angiologie | Münster, DE | 2 |
| Christian Butter | Immanuel Hospital Herzzentrum Brandenburg | Bernau, DE | 1 |
|  |  |  | **Total: 500** |

## Supplementary Table 3. Baseline Demographics and Clinical Characteristics

| **Variable** | **ITT Population (N=500)** |
| --- | --- |
| Age (yr) | 81.8±5.1 (500) |
| Gender, female (%) | 61.2% (306/500) |
| Weight (kg) | 75.5±14.1 (499) |
| Height (cm) | 165.3±8.8 (500) |
| BMI (kg/m^2^) | 27.6±4.8 (499) |
| EuroSCORE II (%) | 6.2±6.8 (457) |
| STS Score (%) | 6.0±4.5 (357) |
| **Pre-existing Clinical Conditions** |  |
| Congestive Heart Failure | 9.4% (47/500) |
| Chronic Obstructive Pulmonary Disease | 11.2% (56/500) |
| Coronary Artery Disease | 53.8% (269/500) |
| Diabetes | 33.4% (167/500) |
| Dyslipidemia | 40.8% (204/500) |
| Hypertension | 88.0% (440/500) |
| Myocardial Infarction | 10.4% (52/500) |
| Peripheral Vascular Disease | 5.8% (29/500) |
| Renal Failure | 30.2% (151/500) |
| Renal Failure requiring hemodialysis | 1.6% (8/500) |
| Stroke | 7.4% (37/500) |
| Transient Ischemic Attack | 4.4% (22/500) |
| **Previous Cardiovascular Interventions** |  |
| Coronary Artery Bypass Graft | 8.0% (40/500) |
| Percutaneous Coronary Intervention | 13.4% (67/500) |
| Pacemaker/Defibrillator Implantation | 11.4% (57/500) |
| Peripheral Interventions | 2.2% (11/500) |
| **Aortic Leaflet Calcification (Site-reported CT data)** |  |
| None | 0.7% (3/426) |
| Mild | 20.0% (85/426) |
| Moderate | 43.2% (184/426) |
| Severe or Extreme | 36.2% (154/426) |

Data are % (n/N) or mean ± standard deviation (n).

## Supplementary Figure 1. Prosthetic Valve Cover Index

A post-hoc analysis compared valve cover index [CI = 100 x (nominal prosthesis diameter – annulus diameter) / nominal prosthesis diameter] in patients with no/trace paravalvular leak (PVL) vs mild or greater PVL at hospital discharge. P-valve is derived from a two-tailed Student t-test.


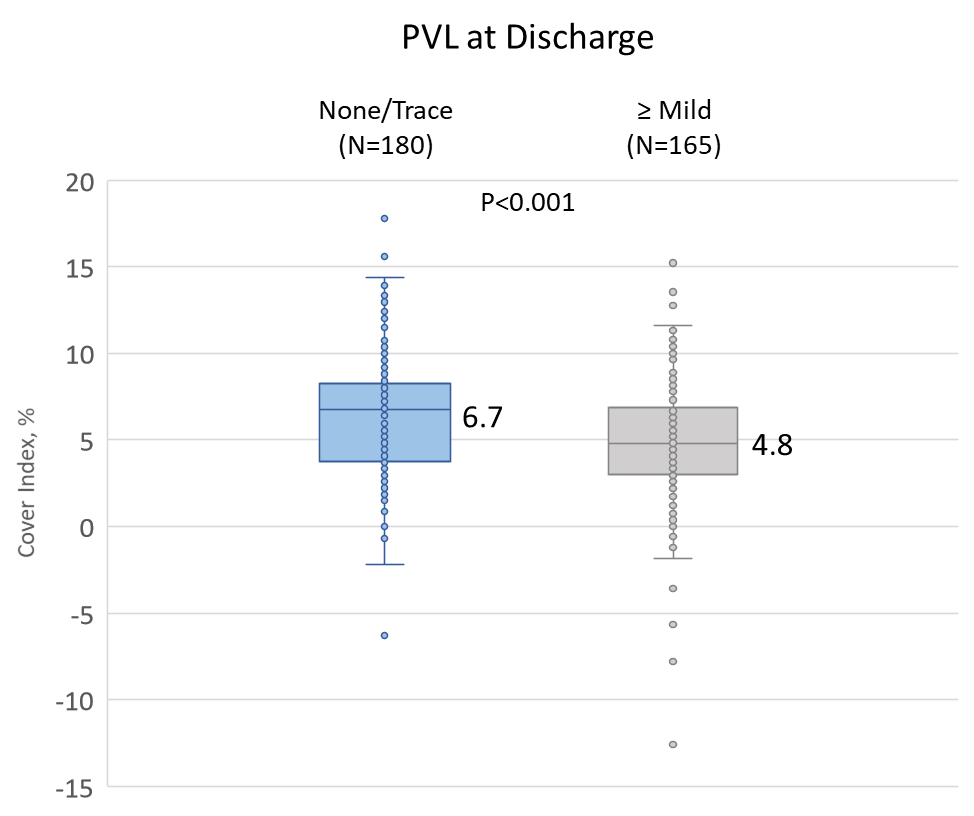


## Supplementary Figure 2. Hemodynamic Results by Valve Size

Core laboratory assessment of echocardiographic data indicates improvements in mean aortic valve (AV) gradient and mean effective orifice area (EOA) through 1 year for all implanted sizes of the valve.


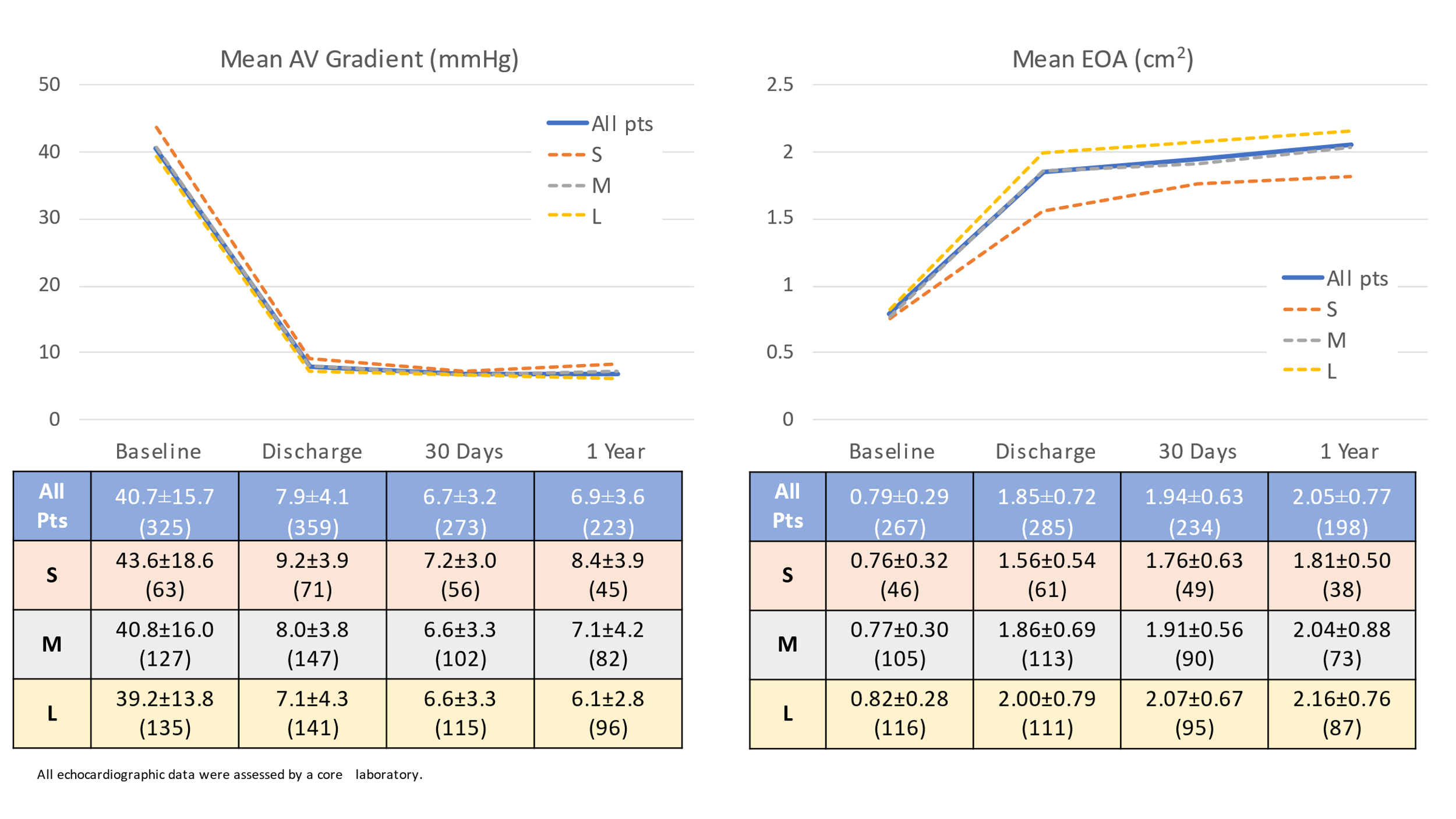

Supplement: Supplementary Materials — Supplementary Table 1: primary and secondary endpoints. Supplementary Table 2: investigators and sites by enrolment. Supplementary Table 3: baseline demographics and clinical characteristics. Supplementary Figure 1: prosthetic valve cover index. Supplementary Figure 2: hemodynamic results by valve size. [file 9138403.f1.docx]
